# Glucocorticoid Metabolism in Hypertensive Disorders of Pregnancy: Analysis of Plasma and Urinary Cortisol and Cortisone

**DOI:** 10.1371/journal.pone.0144343

**Published:** 2015-12-04

**Authors:** Katarzyna Kosicka, Anna Siemiątkowska, Mariola Krzyścin, Grzegorz H. Bręborowicz, Matylda Resztak, Aleksandra Majchrzak-Celińska, Marek Chuchracki, Franciszek K. Główka

**Affiliations:** 1 Department of Physical Pharmacy and Pharmacokinetics, Poznan University of Medical Sciences, Poznań, Poland; 2 Department of Perinatology and Gynecology, Poznan University of Medical Sciences, Poznań, Poland; 3 Department of Pharmaceutical Biochemistry, Poznan University of Medical Sciences, Poznań, Poland; University of Berne, SWITZERLAND

## Abstract

**Objectives:**

The aim of the study was to analyze the plasma and urinary cortisol (F) and cortisone (E) levels in normotensive and hypertensive pregnant women. The parameters known to reflect the function of 11β-hydroxysteroid dehydrogenase type 2 (11β-HSD2) were calculated to verify the changes in glucocorticoid balance over the course of gestational hypertension (GH) and pre-eclampsia (PE).

**Materials and Methods:**

This retrospective case-control study included women in the third trimester of pregnancy, diagnosed with: GH (n = 29), PE (n = 26), or chronic hypertension (CH; n = 22). Normotensive women in their third trimester of pregnancy were also included (controls; n = 43). The plasma and urinary F and E levels were measured with the HPLC-FLD method. The 11β-HSD2 function was estimated by calculating the following ratios: plasma F/E and urinary free F to urinary free E (UFF/UFE). A statistical analysis was performed based on case-control structure.

**Results and Discussion:**

PE was characterized by lower plasma F levels (639.0 nmol/L), UFF/Cr levels (3.80 μg/mmol) and F/E ratio (3.46) compared with that of the controls (811.7 nmol/L, 6.28 μg/mmol and 5.19, respectively) with marked abnormalities observed in the changes of F/E and UFF/UFE ratios with advancing gestation. GH patients showed significant disparities in the urinary steroid profile with lower UFF/UFE ratio (0.330 vs. 0.401) compared with the normotensive controls and abnormal changes in the UFF/UFE throughout pregnancy. The observed tendency towards lower F/E and UFF/UFE ratios in PE and GH patients may reflect more intensive F metabolism over the course of those disorders. In the normal pregnancy group, the plasma F/E and UFF/UFE ratios tended to present inverse correlations with advancing gestation. This trend was much less marked in PE and GH patients, suggesting that the abnormalities in 11β-HSD2 functions progressed with the GA. The birth weights of neonates born from pre-eclamptic pregnancies were lower than those from uncomplicated pregnancies, although only when the babies were born prematurely. Children born at term to normotensive mothers or mothers suffering from PE had comparable birth weights.

## Introduction

Hypertensive disorders of pregnancy (HDsP) complicate up to 10% of pregnancies and are among the most common causes of poor perinatal outcomes [1–3]. These disorders significantly increase the risk of pre-term deliveries, placental abruptions and maternal and neonatal deaths. HDsP are also associated with a higher percentage of cardiovascular and metabolic disorders in the mother and her offspring many years after hypertensive pregnancy [2]. HDsP consist of several diseases and conditions in pregnant women that are characterized by elevated arterial blood pressure, including pre-eclampsia (PE), eclampsia, gestational hypertension (GH) and chronic hypertension (CH) [3]. Despite years of research and a number of hypotheses, the causes of GH and PE remain elusive [4].

11β-hydroxysteroid dehydrogenase type 2 (11β-HSD2) is mainly expressed in the renal tubules and converts cortisol (F) to inactive cortisone (E), thus allowing the mineralocorticoid receptor to be aldosterone-selective [5,6]. The diminished function of this enzyme is caused by various mutations in the *HSD11B2* gene, which encodes 11β-HSD2, and it is responsible for an inherited form of hypertension known as apparent mineralocorticoid excess (AME). The syndrome is characterized by excessive F, which begins to interact with the mineralocorticoid receptor and results in sodium and water retention and eventually causes hypervolemic hypertension [7]. In pregnancy, this enzyme provides an additional function because it is present in the placenta; thus, it protects the baby from the deleterious effects of maternal glucocorticoids (GCs) whose concentrations in pregnant women are several times higher than in the developing fetus. During gestation, the efficient pre-receptor metabolism of GCs is necessary because F, which is essential in small quantities for fetal organ maturation, exerts a proapoptotic effect on the child when it occurs in excess [6,8]. Among many theories, the impaired function of placental 11β-HSD2 has been suggested to explain the etiopathogenesis of GH and PE [5]. Supporting this 11β-HSD2 hypothesis, children born from pre-eclamptic pregnancies are usually smaller than children born from normotensive pregnancies, and this decrease in size may have been caused by an excess of F in utero [6,8,9]. Moreover, women with a proven defect in 11β-HSD2 (suffering from AME) and mice lacking 11β-HSD2 are more prone to pre-term deliveries, miscarriages and low birth weight, which is similar to the outcomes in PE patients [2,7,10]. Decreased 11β-HSD2 activity in the placental tissue was found over the course of both GH and PE [9,11–13]. Nevertheless, this abnormal function should also be evident in maternal matrices; however, reports on this subject are contradictory [14–17].

Therefore, this study aimed to measure and compare the plasma and urinary F and E levels in normotensive and hypertensive women in the third trimester of pregnancy to estimate the function of 11β-HSD2, the pivotal enzyme for GC metabolism. The function of 11β-HSD2 was estimated by calculating two parameters: the F/E ratio in plasma and the urinary free F to urinary free E (UFF/UFE), which have been described as good predictors of the catalytic activity of this enzyme [18].

## Materials and Methods

### Study groups

Subjects were recruited from inpatient populations from the Wielkopolska region who were treated at the Gynecological and Obstetrics University Hospital of Poznan University of Medical Sciences (Poland). The study included 120 women aged 17–44 with singleton pregnancy in the third trimester (27–41 weeks of gestation, WG). The exclusion criteria included stillbirth, endocrine diseases (except for hypothyroidism), liver disease, mental disorders and chronic infectious diseases. The study protocol complied with the Declaration of Helsinki and was approved by the Ethical Committee at Poznan University of Medical Sciences. Written informed consent was obtained from all participants.

The patients were divided into 4 groups as shown in Table 1. GH was diagnosed in women who had not suffered from hypertension before pregnancy and subsequently developed it after 20 WG, whereas CH was diagnosed in patients with pre-pregnancy hypertension or with hypertension developed before 20 WG. According to the WHO [3], PE was defined as an episode of hypertension (with consistent blood pressure ≥140/90 mmHg) and newly onset proteinuria >0.3g/24h. The control group consisted of normotensive pregnant women without proteinuria who fulfilled the inclusion criteria and did not develop intrauterine growth restriction (IUGR). IUGR was defined as birth weight below the tenth percentile for the gestational age (GA) and gender according to data from the Wielkopolska region [19]. Prematurity was defined as giving birth prior to 37 WG. Hypertensive subjects were treated with antihypertensive agents that are recommended for use during pregnancy (primarily with methyldopa or a combination of methyldopa and other drugs). One GH asthmatic patient was undergoing chronic therapy with inhaled GCs (budesonide). The detailed characteristics of the study subjects are shown in Table 1.

**Table 1:** Characteristics of the patients: matching criteria, concomitant diseases, and medication use.

	control	GH	PE	CH
	*(n = 43)*	*(n = 29)*	*(n = 26)*	*(n = 22)*
**Age** (y)^a^	30.7±4.9	30.8±4.5	31.4±6.4	32.3±6.5
**Weight before pregnancy** (kg)^b^	58.0 (53.0–73.0)	74.0 (65.0–81.0)^3^	67.0 (59.5–75.5)	73.5 (57.0–92.0)^1^
**BMI before pregnancy** (kg/m^2^)^b^ ^,^ ^d^	21.2 (19.4–25.6)	25.8 (23.3–29.4)^3^	24.4 (21.7–27.6)^1^	27.1 (22.6–32.2)^2^
• BMI ≥ 25^c^	10 (26.3%)	13 (61.9%)^1^	8 (42.1%)	10 (55.6%)^1^
**Nulliparity** ^c^	14 (32.6%)	15 (51.7%)	15 (57.7%)^1^	12 (54.6%)
**Concomitant diseases**:				
• hypothyroidism^c^	4 (9.3%)	6 (20.7%)	1 (3.8%)	3 (13.6%)
• gestational diabetes^c^	3 (7.0%)	4 (13.8%)	3 (11.5%)	3 (13.6%)
**GA at sample collection** (wks)^b^	37 (36–39)	38 (35–39)	33 (30–36)^3^	37 (34–38)
**GA at delivery** (wks)^b^	39 (38–40)	39 (38–39)	35 (33–37)^3^	39 (38–39)
**Prematurity** ^c^	4 (9.8%)	4 (13.8%)	18 (69.2%)^3^	2 (9.5%)
**Birth weight** (g)^b^	3250 (2870–3620)	3140 (2830–3530)	1750 (1465–2650)^3^	3465 (3040–3755)
• prematurely born	2625±492	2198±958	1630±480^1^	3020±990
• full-term	3359±504	3303±579	2988±796	3429±716
**IUGR** ^c^	0 (0.0%)	6 (20.7%)^2^	14 (53.8%)^3^	3 (15.0%)^1^
**Female fetus** ^c^	15 (36.6%)	13 (44.8%)	12 (46.2%)	11 (52.4%)
**Drug intake:**				
• methyldopa ^c^	0 (0.0%)	25 (86.2%)	24 (96.0%)	19 (84.4%)
• metoprolol ^c^	2 (4.9%)	2 (6.9%)	8 (32.0%)	3 (13.6%)
• nitrendypine ^c^	1 (2.4%)	3 (10.3%)	14 (56.0%)	5 (22.7%)
• verapamil ^c^	6 (14.6%)	4 (13.8%)	1 (4.0%)	2 (9.1%)
• magnesium sulfate ^c^	0 (0.0%)	2 (6.9%)	9 (36.0%)	0 (0.0%)

GA–gestational age; GH–gestational hypertension; PE–pre-eclampsia; CH–chronic hypertension

^a^ values presented as mean ± SD

^b ^values presented as median (interquartile range)

^c^ values presented as n (%)

^d ^BMI calculated as weight (kg) divided by the square of height (m)

^1^ p<0.05

^2 ^p<0.005

^3^ p<0.0005.

### Sample collection

Each woman was required to provide one blood and one urine sample from 24-hour urine collection. Blood samples were obtained from 118 women, whereas urine samples were obtained from 114 patients.

#### Plasma

Maternal blood samples were drawn in the morning (7.00–8.00 a.m.) when F secretion is highest because of circadian rhythm of F secretion [20]. The blood samples were centrifuged and plasma aliquots of approximately 1.5 ml were separated and stored at -25°C until further analysis.

#### Urine

Approximately 20 ml of 24-hour urine collection was collected and stored at -25°C until further analysis. The total volume of the 24-hour urine collection was recorded and used to calculate the excreted UFF and UFE. The creatinine content was determined (commercial kit based on the Jaffe method; BioMaxima, Lublin, Poland) and used to confirm that the urine collection data were correct. The UFF and UFE data are expressed as μg of steroid hormone per mmol of urinary creatinine.

Additionally, each patient underwent routine diagnostic tests, including a broader hormonal profile as presented in Table 2.

**Table 2:** Biochemical and hormonal profiles.

	control	GH	PE	CH
**plasma Na** ^**+**^ ^a^	135.8±2.4	135.1±2.4	135.5±4.2	136.2±2.2
(mmol/L)				
**plasma K** ^**+**^ ^b^	4.15	4.20	4.50	4.20
(mmol/L)	(3.90–4.75)	(4.05–4.35)	(4.40–4.75)	(4.10–4.60)
**plasma Cl** ^-^ ^a^	101.4±3.5	102.1±2.2	102.8±3.4	101.4±2.8
(mmol/L)				
**plasma urea** ^b^	14.9	20.3	26.2*	16.6
(mg/dL)	(13.2–20.4)	(16.7–26.2)	(19.3–41.6)	(13.90–21.6)
**urinary Cr** ^b^	9.05	10.02	9.06	9.03
(mmol/24 h)	(6.84–10.14)	(8.01–10.70)	(8.42–11.73)	(7.54–13.52)
**serum progesterone** ^b^ ^,^ ^#^	330.4	366.3	310.2	319.7
	(217.6–471.4)	(246.7–438.2)	(186.8–457.0)	(224.1–391.4)
[ng/mL]				
**serum CRH** ^a^ ^,^ ^#^	7924.6±1879.5	8686.3±1917.3	6999.8±2244.7	7874.8±2158.3
[pg/mL]				
**plasma F** ^b^	811.7	741.6	639.0*	717.3
(nmol/L)	(688.3–943.0)	(672.6–1000.6)	(505.2–744.6)	(583.6–959.4)
**plasma E** ^b^	171.3	188.6	168.0	157.0
(nmol/L)	(134.0–200.8)	(146.2–221.3)	(143.7–251.4)	(141.4–185.2)
**plasma F/E** ^b^	5.19	4.03	3.46*	4.84
	(3.78–5.95)	(3.59–5.38)	(2.66–4.11)	(3.47–6.12)
**UFF/Cr** ^b^	6.28	5.44	3.80*	5.52
(μg/mmol)	(5.06–7.46)	(3.51–6.97)	(3.47–5.80)	(3.20–8.35)
**UFE/Cr** ^b^	14.55	18.03	12.36	15.29
(μg/mmol)	(10.25–18.44)	(11.93–23.25)	(9.32–16.06)	(9.52–19.85)
**UFF/UFE** ^b^	0.401	0.330*	0.351	0.382
	(0.319–0.639)	(0.238–0.446)	(0.273–0.482)	(0.306–0.506)
**urine volume** ^b^	1435	1900*	1645	1635
(mL)	(940–1800)	(1100–2590)	(1175–2375)	(1035–2313)

^a ^values presented as the mean±SD

^b^ values presented as the median (interquartile range)

* p<0.05

^#^ serum progesterone determined using electrochemiluminescence immunoassay (Roche Diagnostics International Ltd., Rotkreuz, Switzerland); serum CRH determined using ELISA method (EIAab Science, Wuhan, China).

### Patient medical history questionnaire

Each woman was required to complete the questionnaire on her general health. The patients were asked to list their pre-pregnancy chronic diseases, any drugs they were currently taking, any illnesses diagnosed during pregnancy and the associated pharmacological treatment, and any herbal products they were taking during this period. Additional questions covered the following subjects: grapefruit juice intake, height, pre-pregnancy weight, weight during the study, WG during the study and information on previous pregnancies (number and any complications).

### F and E measured in the plasma and urine

The assessed F and E plasma concentrations were determined from the total plasma levels, which included free and protein-bound steroids. The levels of F and E in the urine only reflected the free (unconjugated) forms of F and E. The total amounts of UFF and UFE excreted per day were calculated based on the 24-hour urine collection volumes.

A previously described HPLC-FLD method was modified to determine the plasma and urinary F and E levels [21]. One such modification consisted of the use of a modern core-shell Kinetex 5μm XB-C18 100A column (100 x 4.6 mm) instead of a Chromolith RP-18e, which improved the separation and decreased the time required for an analytical run. The HPLC conditions were unchanged, except for the column temperature, which was fixed at 60°C. The detection limit (10 ng/mL) was the same for F and E. The modified method has been validated according to the guidelines of European Medicine Agency and met the requirements for analytical methods.

### Function of 11β-HSD2

Two ratios, the total plasma F to total plasma E (both in nmol/L) and UFF to UFE (both expressed as µg/mmol creatinine), were calculated to assess the function of 11β-HSD2.

### Statistics

The data were analyzed with a case-control model using the Statistica 10 software (StatSoft Inc., Tulsa, OK, USA). After testing the normal distribution (Shapiro-Wilk test), differences in continuous variables between the groups were estimated using a Mann-Whitney U test or ANOVA with post-hoc Tukey’s test (for non-parametric and normally distributed data, respectively), and the results are expressed as medians values (interquartile range) or mean±SD. Disparities in the birth weight between the GH, PE and control patients among premature and full-term babies were analyzed using an ANOVA with a post-hoc Tukey’s test (results expressed as the mean±SD). Differences in the categorical data were analyzed using Fisher’s exact test. A Spearman test was applied to estimate simple correlations between the steroids levels and GA at sampling as well as the steroid levels and CRH concentrations. A one-way logistic regression analysis was performed to assess the influence of maternal factors on the hypertensive disorders during pregnancy development. The results are expressed as odds ratios (ORs) with 95% confidence intervals (95% CIs).

A multiple regression analysis was performed to particularize the data from Mann-Whitney U test to confirm that the disparities in GC profiles are caused by specific complications of pregnancy and do not represent factors that were previously found to differ among groups. The created models were considered significant at p<0.05.

First, we attempted to build models with confounders by applying a stepwise regression method with forward selection (selection F = 1.00; elimination F = 0). We included factors that are suspected or known to determine the GC profile: maternal age, pre-pregnancy BMI, gender of the fetus, nulliparity, thyroid dysfunction, gestational diabetes, IUGR and HDsP (three analyses with PE, GH or CH as the influencing factor).

Second, the impact of the GA at sampling on the GC levels was analyzed in all studied groups, based on reported changes in the steroid profile with advancing gestation [14]. Models of particular GCs (F, E, UFF/Cr or UFE/Cr) or the ratios (F/E or UFF/UFE) as well as the GA at sampling and the appropriate disorder (PE, GH or CH) were built.

A multivariate regression analysis was also performed to assess correlations between the birth weight and GC levels. Unless otherwise noted, a p-value of <0.05 was considered significant.

## Results

### Plasma and urinary GCs and the function of 11β-HSD2

The level of GCs in the plasma and urine and the calculated F/E and UFF/UFE ratios are summarized in Table 2. Samples from one PE and one GH patient had to be excluded from further analyses because the obtained concentrations were below the quantitation limit. Ultimately, steroid profiles were obtained from 116 plasma and 112 urine samples.

A woman receiving inhaled GC therapy had steroid values similar to those of the other GH patients despite reports indicating that this treatment can suppress the plasma F [22]. Therefore, this patient was not excluded from further analyses.

We observed a positive correlation between the GA at sampling and the plasma F and E in the control group (R = 0.310, p = 0.046 and R = 0.421, p = 0.005, respectively); however, the F/E ratio was found to be independent on the GA. This relationship was not clear in hypertensive women, and significant dependence was only observed between the plasma E level and GA in the GH group (R = 0.389, p = 0.037). The urinary GCs did not correlate with the GA in the control and study groups.

A Spearman test confirmed the expected relationship (significant positive correlation) between the levels of serum corticotropin releasing hormone (CRH) and plasma F in all groups.

### Logistic regression analysis

The likelihood of developing GH was directly correlated with the pre-pregnancy weight (p = 0.002, OR = 1.084, 95% CI 1.030–1.141) or BMI value (p = 0.001, OR = 1.387, 95% CI 1.147–1.676). In overweight or obese women (BMI≥25), the risk of GH was over 2 times higher (p = 0.009, OR = 2.133, 95% CI 1.207–3.770) than in women of normal weight. An increase in the pre-pregnancy BMI value by one unit also slightly increased the risk of PE (p = 0.023, OR = 1.200, 95%CI 1.026–1.403). Primiparous women were at a higher risk of suffering from PE (p = 0.043, OR = 1.681, 95% CI 1.016–2.779), and this tendency was observed in relation to both GH (p = 0.107, OR = 1.490, 95% CI 0.918–2.417) and CH (p = 0.090, OR = 1.577, 95% CI 0.931–2.671).

The strongest risk factors for CH were a higher pre-pregnancy weight (p = 0.005, OR = 1.057, 95% CI 1.017–1.098) and BMI (p = 0.003, OR = 1.234, 95% CI 1.074–1.418), particularly a BMI≥25 (p = 0.037, OR = 1.871, 95% CI 1.039–3.370).

### Multiple regression analysis

We found that the PE was associated with a significantly lower plasma F/E ratio (p<0.001, semi-correlation R = -0.466) and a higher maternal pre-pregnancy BMI (p = 0.048, R = 0.247). Furthermore, the plasma F (p = 0.076, R = -0.223) and UFF/UFE ratio (p = 0.061, R = -0.251) tended to be lower in pre-eclamptic patients. A significant relationship was observed between GH and the UFE/Cr value (p = 0.014, R = 0.320), with the following significant confounders: hypothyroidism (p = 0.004, R = 0.378) and nulliparity (p = 0.038, R = -0.267).

**Fig 1:**
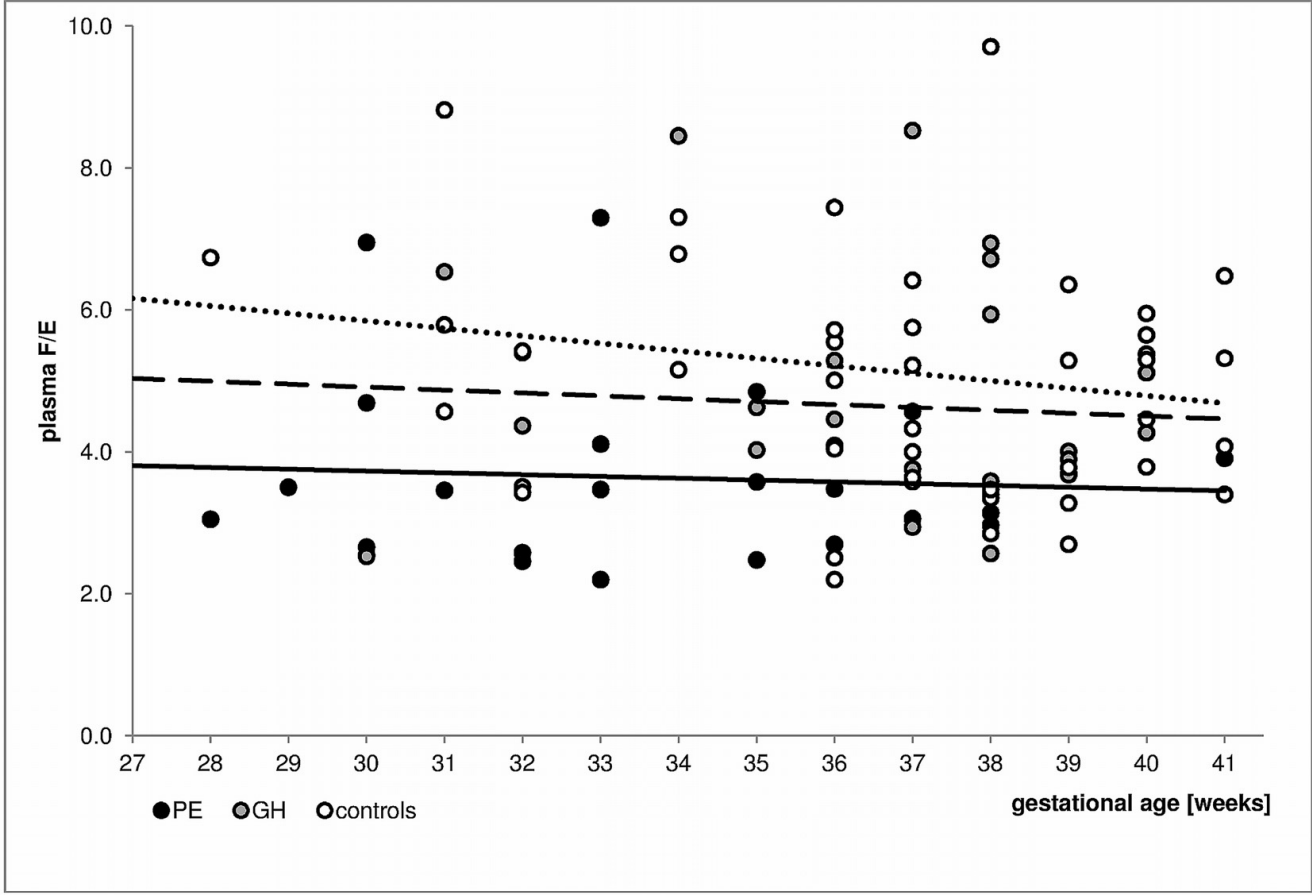
Changes in plasma F/E values by gestational age in the PE, GH and normotensive subjects. The trend lines are marked as follows: the solid line reflects data for the PE subjects, the dashed line represents the data for the GH subjects, and the dotted line represents data for the normotensive patients. The applied multiple regression models showed that PE (p<0.001, R = -0.416) but not the GA (p = 0.250, R = -0.138) significantly influenced the F/E. The F/E ratio was not influenced by neither GH nor the GA.

**Fig 2:**
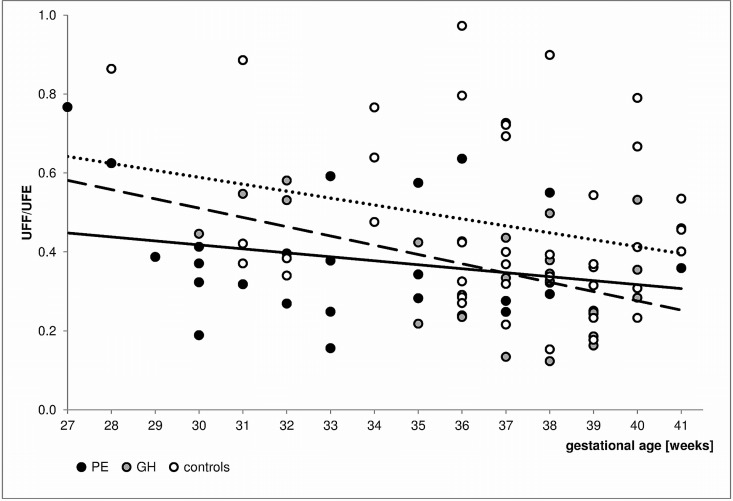
Changes in UFF/UFE values by gestational age in the PE, GH and normotensive subjects. The trend lines are marked as follows: the solid line reflects data for the PE subjects, the dashed line represents data for the GH subjects, and the dotted line represents data for the normotensive patients. The applied multiple regression models indicated that the UFF/UFE was significantly influenced by the PE (p = 0.018, R = -0.290) and nearly significantly by the GA (p = 0.052, R = -0.237). Moreover, the UFF/UFE was significantly dependent on both the GH and GA (p = 0.014, R = -0.287 and p = 0.013, R = -0.290, respectively).

The GCs levels were then analyzed using models that included hypertensive disease and GA at sampling. The plasma E level was significantly positively correlated with both PE (p = 0.003, R = 0.362) and GA at sampling (p = 0.006, R = 0.335). Similar relationships were found between GH (p = 0.045, R = 0.227) and GA (p = 0.002, R = 0.355). The UFF/Cr value was significantly influenced by PE (p<0.001, R = -0.406) and GA (p = 0.014, R = -0.289). The results of the analysis of ratios reflecting the function of 11β-HSD2 are presented as F/E in Fig 1 and UFF/UFE in Fig 2.

After adjusting for the GA at delivery, pre-pregnancy BMI, gestational diabetes and infant's gender, the birth weight was found to be dependent on the total F (p = 0.010, R = -0.159) and total E levels (p = 0.002, R = -0.189). Moreover, the birth weight was negatively correlated (p = 0.006, R = -0.169) with the PE, after controlling for the GA at delivery (p<0.001, R = 0.566). A multivariate regression showed that babies of women with PE are approximately 400 g lighter than normal pregnancy neonates (after adjusting for the GA at delivery). Applying this analysis to the group of prematurely born neonates yielded an even stronger relationship and showed that babies from PE pregnancies tended to be 750 g lighter than normal pregnancy neonates (p = 0.002). PE has not been shown to influence the birth weight of full-term neonates (Table 1).

## Discussion

This study demonstrated differences in the metabolism of GCs between hypertensive and normotensive pregnancies. The identified variations were unexpected because decreased placental 11β-HSD2 activity and expression have been reported over the course of PE [9,12,13]. Because the results for the CH patients presented the greatest similarity with those of normotensive individuals and this type of hypertension is not pregnancy related, the results related to the CH group are discussed separately.

The levels of total plasma F in pregnant women reported by various authors are divergent [14–16,20,23–25] and ranged from approximately 500 nmol/L [14,24] to 1400 nmol/L [23] and 1700 nmol/L [25]. The results obtained in this study are comparable with previously published data. The discrepancies in the F and E levels between various studies may be explained by differences in the GA at sample collection (because the levels of F change throughout pregnancy [14]) or sampling time (not mentioned in most of the studies). Notably, we observed large interindividual diversity between plasma GCs obtained from clinically similar subjects (Table 2) and attributed this difference to between-patient variability in placental 11β-HSD2 function as reported by other authors [26,27].

The plasma F levels were significantly lower in PE patients compared with the controls (Table 2). Although this finding may be confounded by the significantly earlier GA at sampling in the PE patients (the level of F is known to directly correlate with pregnancy term [14,28]), comparing the trend lines of the plasma F levels in relation to the GA (S1 Fig) indicates that the line for PE is clearly below the line for the controls (F values for PE patients are consistently lower). Moreover, both lines are close to parallel, suggesting that the tendency for the plasma F level to progressively increase along with the progression of a normal pregnancy is maintained in PE patients. The results from the multiple regression analysis that only considered the GA as a confounder demonstrated the F levels tended to decrease in PE patients (p = 0.083, R = -0.207). The relationship between the GA and the plasma F level in this analysis was not significant (p = 0.102, R = 0.195).

The plasma F levels did not significantly differ between the GH and normotensive pregnant women, and these results corroborate data presented by other authors [16,24]. However, published reports are unclear and equivocal. For example, the maternal F level has been shown to be higher in uncomplicated pregnancies compared with that in GH and PE patients, and its concentration has been inversely correlated with hypertension severity [14,15]. Our results show a similar trend, although only for PE patients. Discrepancies related to this topic may be caused by differences in the sampling time because F secretion is characterized by a variable circadian rhythm, which is maintained in pregnancy [20,29].

Conversely, we revealed that changes in the plasma E levels with advancing gestation are interrupted in both PE and GH patients. This finding may indicate differences in the 11β-HSD2 function, which is confirmed by the reduced F/E ratio among pre-eclamptic subjects and the tendency towards lower F/E values in GH patients (Fig 1). The decreased plasma F/E ratios with advancing gestation observed in normal pregnancy may reflect the reported [27] increase in placental 11β-HSD2 activity with gestation. A limitation of this study is a lack of data concerning F/E ratios in newborns (assessed in umbilical cord vein). Therefore, the extent to which the maternal F/E ratio only reflects the function of 11β-HSD2 in the placenta and not in other tissues cannot be precisely determined [25]. However, the inverse relationship between the F/E and GA was much less marked in the PE and GH patients compared with that of the normotensive group, suggesting that the abnormalities in the overall 11β-HSD2 activity progressed along with GA. Moreover, the trend lines for both GH and PE were below that of the controls. Unexpectedly, this finding may suggest more intensive F metabolism over the course of those disorders. Furthermore, the assumption that plasma F/E reflects the 11β-HSD2 function may not be accurate. The impact of 11β-HSD type 1, which converts E to F, on GC equilibrium must be considered. Specifically, polymorphisms in the *HSD11B1* gene (the gene encoding 11β-HSD1) have been associated with PE development, and variations in *HSD11B1* have been reported as potential biomarkers in disease diagnosis [30].

The dependences observed in the plasma steroids were reflected in the urinary steroid profile. Similar to that of the plasma F levels, significant disparities in the UFF/Cr were found between the PE and control groups (Mann-Whitney U test, Table 2) but not between the GH and control groups. The relationship for PE remained significant after controlling for the GA (p<0.001, R = -0.406 for PE and p = 0.014, R = -0.289 for the GA). Previously published studies present confusing observations on the urinary GCs between normotensive subjects and patients with pregnancy related hypertension (with or without proteinuria). According to the urinary F levels, our conclusions are partially similar to those of *Walker et al*. [16] (differences in UFF were not observed between the GH and control subjects), which are inconsistent with the data presented by *Heilmann et al*. [17]. These discrepancies may have been caused by a lack of GC correction by creatinine in the cited studies. Conversely, we observed similar levels of UFE/Cr in all studied groups, which corroborates reports in the literature [16,17].

The UFF/UFE ratio, which is considered to provide the most accurate indicator of 11β-HSD2 activity, was significantly lower (Table 2) in the GH patients. The multivariate analysis indicated a significant or nearly significant association between both the GA at sampling and GH or PE and UFF/UFE values. The UFF/UFE was inversely correlated with gestation and significantly lower in the GH and PE subjects (Fig 2). F metabolism was more intensive in the GH patients (lower UFF/UFE values), although the trend observed in the control group was maintained in the GH group. However, the trend line for the PE patients was markedly different from that of the control or GH patients, which suggests significantly altered F metabolism in the PE subjects. In our opinion, the previously reported increase in placental 11β-HSD2 function with pregnancy progression [27] was reflected in our analysis of the urinary steroid profile. Moreover, the cortisol-cortisone equilibrium was clearly interrupted over the course of PE, and the conspicuous increase in F metabolism was absent in the pregnancies approaching full term.

Patients with CH did not present significant disparities in steroid metabolism. Specifically, neither the Mann Whitney U test nor the multiple regression analysis revealed differences between the CH patients and controls. Although this finding is consistent with other reports concerning the plasma or urinary F and E levels in hypertensive pregnant women [24,31], it is inconsistent with the hypothesized impaired 11β-HSD2 activity in the general CH population [32]. Conversely, this finding may suggest that the described differences are characteristic for pregnancy-specific disorders.

In this study, we confirmed that pre-pregnancy health status significantly influences GH and PE development, and the strongest risk factors included higher weight and BMI (especially BMI≥25, indicating overweight and obese conditions) and nulliparity, which corroborated the findings in the literature [33]. However, we did not observe a correlation between maternal age and these conditions.

We found that maternal plasma GCs were associated with the birth weight of newborns. The weight at birth was negatively correlated with the total F and E levels when controlled for confounders. Lower birth weight of neonates born from pre-eclamptic pregnancies could be caused by early delivery GA. However, we observed a significant negative correlation between neonate weight and PE diagnoses when adjusted for the GA at delivery. This finding is consistent with results in the literature, which indicate that children born from pre-eclamptic pregnancies are smaller than children born from healthy pregnancies [34]. Nevertheless, this phenomenon applies to pre-term neonates and is not significant when babies are born at term [35], which was confirmed in our studies (Table 1).

In conclusion, this preliminary study showed significant differences in F metabolism between normotensive pregnant women and PE or GH patients. The plasma and urinary GC profile evaluations may be used as a tool for estimating the mechanism and consequences of PE as well as GH development. Our main findings include the abnormal changes of 11β-HSD2 function that occur as pregnancies approach full term in pre-eclamptic patients and the significant discrepancies in birth weight between the premature infants in the PE and control group.

## Supporting Information

S1 Fig The changes in plasma F values towards term in the PE, GH and normotensive subjects. The trend lines are marked as follows: the solid line reflects data for the PE subjects, the dashed line represents the data for GH subjects, the dotted line represents data for the normotensive patients. The applied multivariate regression models showed that PE (p = 0.083, R = -0.207) and the GA (p = 0.102, R = 0.195) do not influence significantly plasma F values. Plasma F level did not depend significantly on the GH, but was significantly influenced by the GA (p = 0.043, R = 0.242).
(TIF)
