# Supplementary figures and images for: Glucocorticoid Metabolism in Hypertensive Disorders of Pregnancy: Analysis of Plasma and Urinary Cortisol and Cortisone
Source: PLoS One. 2015 Dec 4;10(12):e0144343. doi: 10.1371/journal.pone.0144343 (PMC4670176; doi:10.1371/journal.pone.0144343)

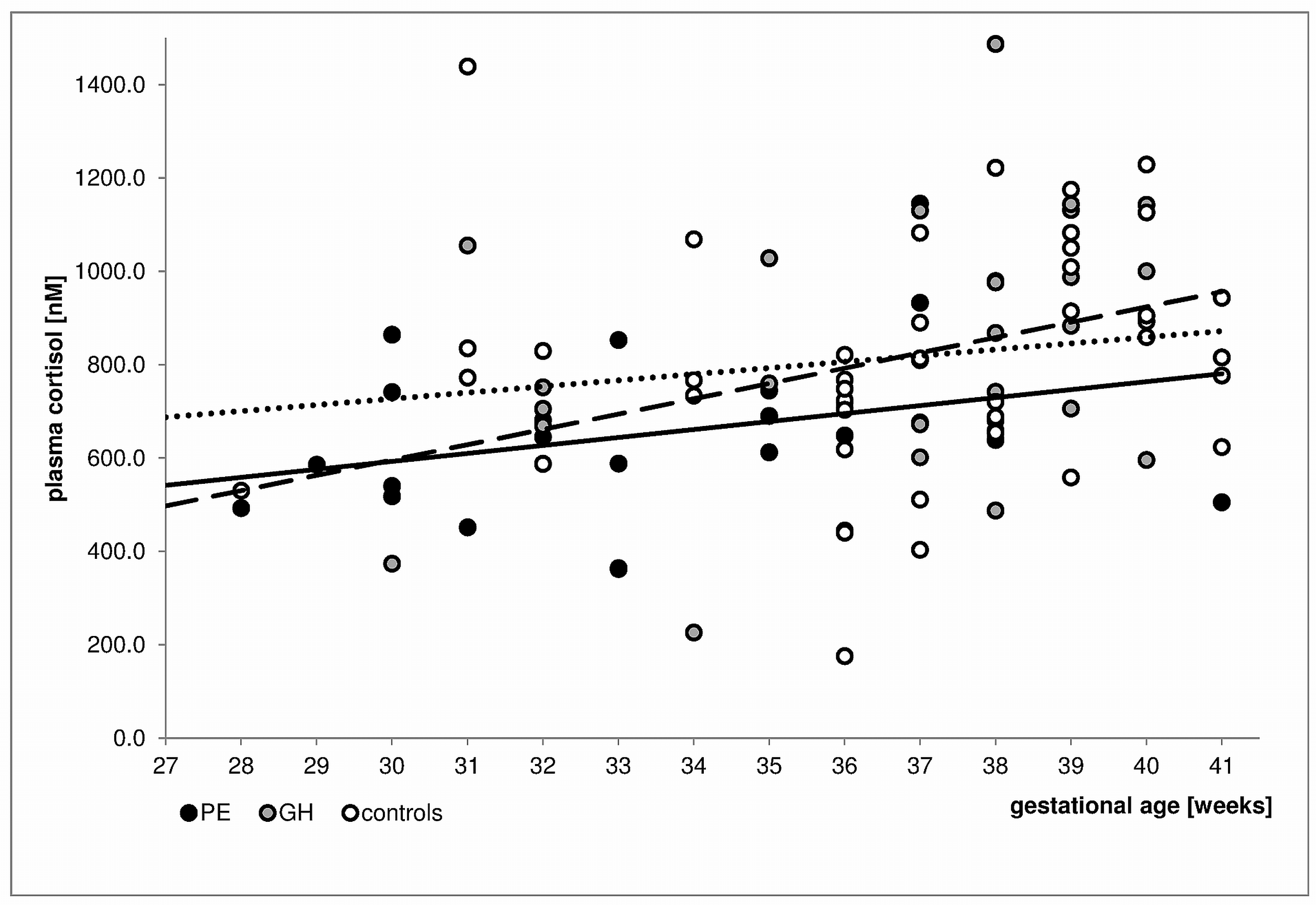

Supplement: S1 Fig — The trend lines are marked as follows: the solid line reflects data for the PE subjects, the dashed line represents the data for GH subjects, the dotted line represents data for the normotensive patients. The applied multivariate regression models showed that PE (p = 0.083, R = -0.207) and the GA (p = 0.102, R = 0.195) do not influence significantly plasma F values. Plasma F level did not depend significantly on the GH, but was significantly influenced by the GA (p = 0.043, R = 0.242). (TIF) [file pone.0144343.s001.tif]
